# OrfM: a fast open reading frame predictor for metagenomic data

**DOI:** 10.1093/bioinformatics/btw241

**Published:** 2016-05-03

**Authors:** Ben J. Woodcroft, Joel A. Boyd, Gene W. Tyson

**Affiliations:** Australian Centre for Ecogenomics, School of Chemistry and Molecular Biosciences, University of Queensland, Brisbane, QLD 4072, Australia

## Abstract

**Summary:** Finding and translating stretches of DNA lacking stop codons is a task common in the analysis of sequence data. However, the computational tools for finding open reading frames are sufficiently slow that they are becoming a bottleneck as the volume of sequence data grows. This computational bottleneck is especially problematic in metagenomics when searching unassembled reads, or screening assembled contigs for genes of interest. Here, we present OrfM, a tool to rapidly identify open reading frames (ORFs) in sequence data by applying the Aho–Corasick algorithm to find regions uninterrupted by stop codons. Benchmarking revealed that OrfM finds identical ORFs to similar tools (‘GetOrf’ and ‘Translate’) but is four-five times faster. While OrfM is sequencing platform-agnostic, it is best suited to large, high quality datasets such as those produced by Illumina sequencers.

**Availability and Implementation:** Source code and binaries are freely available for download at http://github.com/wwood/OrfM or through GNU Guix under the LGPL 3+ license. OrfM is implemented in C and supported on GNU/Linux and OSX.

**Contacts:** b.woodcroft@uq.edu.au

**Supplementary information:** Supplementary data are available at *Bioinformatics* online.

## 1 Introduction

In genomics, stretches of DNA uninterrupted by stop codons are known as open reading frames (ORFs). The TAG (‘amber’), TAA (‘ochre’) and TGA (‘opal’) stop codons signal the ribosomal machinery to cease translation, with few exceptions. An extended stretch of DNA free of in-frame stop codons is evidence that a gene may be encoded on that region.

ORF prediction in metagenomics can be performed on finished population genomes, draft population genomes, assembled contigs or individual reads. Searching for genes in individual metagenomic reads (‘gene-centric analysis’) is useful when reference genomes are unavailable and assembly of reads is either computationally prohibitive or a microbial community is too complex for successful assembly (Howe and Chain, 2015). In long assembled sequences, conventional gene predictors use information such as codon usage to more accurately predict genes, but these signals become unreliable in the limited genomic context of short read data.

In bacterial and archaeal genomes, genes are not interrupted by exons and intergenic space is minimal, so short read sequences derived from these genomes are more likely to encode a fragment of a gene uninterrupted by a stop codon. ORF prediction directly on early next generation sequencing platforms (e.g. Roche 454) was difficult as they produced reads prone to insertion deletion (indel) errors. In contrast, newer Illumina-based sequencers generate reads where indel errors are rare; reads are higher quality and the errors that do occur are chiefly substitution errors (Jünemann *et al.*, 2013). The current widespread use of Illumina sequencing in metagenomics (Bragg and Tyson, 2014) presents an opportunity to find ORFs in microbial reads directly.

Identification of ORFs in short read data simplifies downstream comparative analysis and allows use of tools that require protein sequence as input e.g. searching for protein families with HMMER (Camacho *et al.*, 2009). Using ORFs instead of six-frame translating sequences for downstream sequence comparison tools e.g. BLAST (Camacho *et al.*, 2009) minimizes the impact of multiple hypothesis testing so results may be more significant.

While finding ORFs in short read data provides advantages over gene prediction and six-frame translation, current ORF finders do not scale to the large size of modern metagenomes e.g. He *et al.* (2015), >500 Gb. Here, we present OrfM, a tool to rapidly identify ORFs in metagenomic datasets.

## 2 Inputs and outputs of OrfM

OrfM uses FASTA or FASTQ (gzip-compressed or uncompressed) sequences as input, and can accept other input formats if converted to FASTA and streamed via the UNIX STDIN pipe. OrfM handles these input format files through its use of kseq.h (http://lh3lh3.users.sourceforge.net/kseq.shtml). By default, the minimum ORF length reported by OrfM is set to 96 bp (32 amino acids). This threshold was driven by the current prevalence of 100 bp Illumina HiSeq reads: the 96 bp cutoff is the maximal size of ORF such that a reading frame can be found in each of the 6 reading frames of a 100 bp read. All ORFs greater than the threshold length are reported even if they overlap. As well as the standard translation table, OrfM can use the 18 alternative translation tables. OrfM outputs amino acid FASTA sequences whose header is the same as the input sequence, with the addition of a string ‘_X_Y_Z’ to the first word, where X is the start position, Y is the frame number and Z is the ORF number. This naming scheme allows ORFs to be located in the original sequence and ensures that the names of the ORFs are unique. OrfM can also output the corresponding nucleotide sequences of the ORFs, if desired.

## 3 Algorithm

In contrast with previous methods which first translate the input sequence into 6 frames and then scan through these translated strings looking for stop codons, OrfM identifies stop codons in nucleotide sequences directly, using an Aho–Corasick search dictionary (Aho and Corasick, 1975). Further details can be found in Supplementary Text S1.

## 4 Benchmarking

**Fig. 1.**
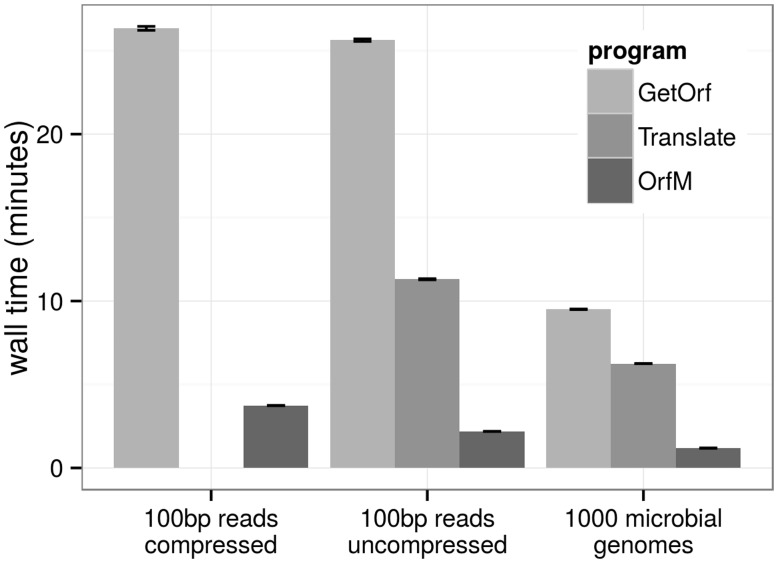
Time taken (wall time) by each program for the benchmark datasets. GetOrf and Translate take significantly more time than OrfM to call ORFs. Translate is unable to run on compressed reads therefore wall time was not measured for the first dataset. Error bars indicate standard error of mean among triplicate runs

OrfM was compared (Supplementary Text S2) with ‘GetOrf’ from the emboss suite (Tringe *et al.*, 2005) (version 6.6.0) and the ‘Translate’ tool from the biosquid package version 1.9g+cvs20050121 (Eddy, unpublished http://eddylab.org/software.html). The tools were compared using three public datasets on a single core of a 20 core 2.3 GHz Intel Xeon E5-2650 running Linux 3.2.0. The benchmark datasets were (i) the forward 100 bp reads of a HiSeq 2000 metagenome (5.5 Gb) (Shakya *et al.*, 2013) in gzip-compressed FASTQ format, (ii) the same reads transformed into uncompressed FASTA format and (iii) a collection of 1000 microbial genomes selected randomly from the Integrated Microbial Genomes (IMG) 4.1 database (Markowitz *et al.*, 2012) in FASTA format (Supplementary Table S1). FASTA sequences converted from compressed FASTQ were streamed into GetOrf using the UNIX STDIN pipe (here using gzip for decompression and awk for conversion to FASTA), while Translate does not accept streamed sequences, so the compressed FASTQ benchmark was not carried out. Translate was run with a minimum ORF size of 32 (-l 31), and GetOrf with a minimum nucleotide size of 96 (-minsize 96) in order to constrain the minimum output ORF length to the default cutoff of OrfM. In all cases OrfM was the fastest, taking 20 and 21% of the time required for translate and GetOrf respectively (Fig. 1). The set of ORFs produced by each of the three methods were identical when reads containing ambiguous nucleotides were omitted from the comparison.

## Supplementary Material

Supplementary Data

## References

[btw241-B1] Aho A.V. Corasick M.J. (1975) Efficient string matching: an aid to bibliographic search. Communications of the ACM, 18, 333–340.

[btw241-B2] Bragg L Tyson G.W. (2014) Metagenomics using next-generation sequencing. Methods Mol. Biol. 1096, 183–201. 24515370 10.1007/978-1-62703-712-9_15

[btw241-B16] Camacho C (2009) BLAST+: architecture and applications. BMC bioinformatics, 10, 421. 20003500 10.1186/1471-2105-10-421 PMC2803857

[btw241-B3] He S (2015) Patterns in wetland microbial community composition and functional gene repertoire associated with methane emissions. mBio, 6, e00066–e00015. 25991679 10.1128/mBio.00066-15 PMC4442139

[btw241-B4] Howe A. Chain P.S. (2015) Challenges and opportunities in understanding microbial communities with metagenome assembly (accompanied by IPython Notebook tutorial). Front. Microbiol., 6. 10.3389/fmicb.2015.00678 PMC4496567 26217314

[btw241-B5] Jünemann S (2013) Updating benchtop sequencing performance comparison. Nat. Biotechnol., 31, 294–296. 23563421 10.1038/nbt.2522

[btw241-B6] Markowitz V.M (2012) IMG: the Integrated Microbial Genomes database and comparative analysis system. Nucleic Acids Res., 40, D115–D122. 22194640 10.1093/nar/gkr1044 PMC3245086

[btw241-B7] Shakya M (2013) Comparative metagenomic and rRNA microbial diversity characterization using archaeal and bacterial synthetic communities. Environ. Microbiol., 15, 1882–1899. 23387867 10.1111/1462-2920.12086 PMC3665634

[btw241-B8] Tringe S.G (2005) Comparative metagenomics of microbial communities. Science, 308, 554–557. 15845853 10.1126/science.1107851

