# Supplementary material for: OrfM: a fast open reading frame predictor for metagenomic data
Source: Bioinformatics. 2016 May 3;32(17):2702–3. doi: 10.1093/bioinformatics/btw241 (PMC5013905; doi:10.1093/bioinformatics/btw241)
Supplement: Supplementary Data [file supp_32_17_2702__index.html]

OrfM: a fast open reading frame predictor for metagenomic data — OrfM: a fast open reading frame predictor for metagenomic data — Supplementary Data 

# OrfM: a fast open reading frame predictor for metagenomic data

## Supplementary Data

files

- Supplementary Data - zip file
