# Supplementary material for: OrfM: a fast open reading frame predictor for metagenomic data
Source: Bioinformatics. 2016 May 3;32(17):2702–3. doi: 10.1093/bioinformatics/btw241 (PMC5013905; doi:10.1093/bioinformatics/btw241)
Supplement: Supplementary Data [file supp_btw241_suppl_data_updated.zip › SupplementaryText2.doc]

OrfM Algorithm

OrfM finds open reading frames in nucleotide sequences using an Aho-Corasick search dictionary (Aho and Corasick, 1975) which includes the codons TAG, TAA and TGA as well as their reverse complements. This data structure is used to scan the sequence in order so that stop codons are found in left to right order through the sequence. When a stop codon is encountered, its position in the sequence and direction are stored. The frame of the ORF is determined with a modulo 3 operation and the length of the ORF is found by comparison with the last found position in that frame (or the sequence boundary). If the ORF is longer than the threshold length, it is translated into amino acids using a lookup table which uses the 3 bases of the codon as a direct index into a table of size 2^3*8^ characters. This simple ASCII codon to amino acid indexing scheme allows the compiler to vectorize the loop that translates each codon. Finding ORFs using alternative translation tables are similarly implemented by using the relevant stop codons to initiate the search dictionary.
